# Profiles of Acute Cytokine and Antibody Responses in Patients Infected with Avian Influenza A H7N9

**DOI:** 10.1371/journal.pone.0101788

**Published:** 2014-07-08

**Authors:** Rui Huang, Lu Zhang, Qin Gu, Yi-Hua Zhou, Yingying Hao, Kui Zhang, Yong Liu, Danjiang Dong, Shixia Wang, Zuhu Huang, Shan Lu, Chao Wu

**Affiliations:** 1 Department of Infectious Diseases, Nanjing Drum Tower Hospital, Nanjing University Medical School, Nanjing, China; 2 Department of Intensive Care Units, Nanjing Drum Tower Hospital, Nanjing University Medical School, Nanjing, China; 3 Department of Laboratory Medicine, Nanjing Drum Tower Hospital, Nanjing University Medical School, Nanjing, China; 4 Jiangsu Key Laboratory for Molecular Medicine, Nanjing Drum Tower Hospital, Nanjing University Medical School, Nanjing, China; 5 Clinical College of Traditional Chinese and Western Medicine, Nanjing University of Chinese Medicine, Nanjing, China; 6 Department of Infectious Diseases, The First Affiliated Hospital of Nanjing Medical University, Nanjing, China; 7 China-US Vaccine Research Center, The First Affiliated Hospital of Nanjing Medical University, Nanjing, China; 8 Department of Medicine, University of Massachusetts Medical School, Worcester, Massachusetts, United States of America; German Primate Center, Germany

## Abstract

The influenza A H7N9 virus outbreak in Eastern China in the spring of 2013 represented a novel, emerging avian influenza transmission to humans. While clinical and microbiological features of H7N9 infection have been reported in the literature, the current study investigated acute cytokine and antibody responses in acute H7N9 infection. Between March 27, 2013 and April 23, 2013, six patients with confirmed H7N9 influenza infection were admitted to Drum Tower Hospital, Nanjing, China. Acute phase serum cytokine profiles were determined using a high-throughput multiplex assay. Daily H7 hemagglutinin (HA)-specific IgG, IgM, and IgA responses were monitored by ELISA. Neutralizing antibodies specific for H7N9 viruses were determined against a pseudotyped virus expressing the novel H7 subtype HA antigen. Five cytokines (IL-6, IP-10, IL-10, IFNγ, and TNFα) were significantly elevated in H7N9-infected patients when compared to healthy volunteers. Serum H7 HA-specific IgG, as well as IgM and IgA responses, were detected within 8 days of disease onset and increased in a similar pattern during acute infection. Neutralizing antibodies developed shortly after the appearance of binding antibody responses and showed similar kinetics as a fraction of the total H7 HA-specific IgG responses. H7N9 infection resulted in hallmark serum cytokine increases, which correlated with fever and disease persistence. The novel finding of simultaneous development of IgG, IgM, and IgA responses in acute H7N9 infection points to the potential for live influenza viruses to elicit fast and potent protective antibodies to limit the infection.

## Introduction

An emerging Type A influenza H7N9 infection in humans, which started in early 2013, has continued in China and represents another major threat to global health [1]–[20]. H7N9 has a mortality rate of 32.4% [21]. Multiple environmental and/or virological changes may have contributed to this outbreak [22], [23]. While the clinical symptoms and features of isolated H7N9 virus strains have recently been described, information on early immune responses in acutely H7N9-infected patients is limited [5]–[7], [24]–[27]. Given the importance of antibody responses in protection immunity against influenza and the role of cytokines in modulating innate immune responses in patients infected with influenza viruses, the current report analyzed serum H7 HA-specific binding antibody responses starting within 6–11 days after onset of fever in H7N9 patients, the development of neutralizing antibodies, and serum levels of specific cytokines in a cohort of six H7N9-infected patients admitted to a hospital in Nanjing during the peak of the 2013 outbreak. Due to limited knowledge in the existing literature regarding acute immune responses to an outbreak of a novel avian influenza in humans, information described in this report may be useful for a better understanding on the development of acquired and innate immunities early after avian influenza infection.

## Materials and Methods

### Patient information and sample collection

Between March 27, 2013 and April 23, 2013, six patients were admitted to the Nanjing Drum Tower Hospital (NDTH) (Table 1) with confirmed influenza H7N9 virus infection via detection of viral RNA with real-time PCR [7]. Sputum and blood samples were collected as part of routine clinical management. Blood samples were collected from ten healthy volunteers (five males and five females; aged 32–59 years) as controls. The study was reviewed and approved by the Ethics Committee at Nanjing Drum Tower Hospital and written informed consent was obtained from each participant or their legal representative.

**Table 1:** Basic characteristics of H7N9-infected patients.

	H7N9-infected patients					
	PT-1	PT-2	PT-3	PT-4	PT-5	PT-6
Age	45	48	61	30	54	36
Gender	F	F	F	M	M	M
Occupation	Poultry worker	Farmer	House wife	Chef	Clerk	Poultry retailer
Poultry contact history	Yes	Yes	Yes	No	No	Yes
Admission (days after fever onset)	9	11	8	8	9	6
Hospital Stay (days)	>60	35	>60	38	28	22

### Influenza H7N9 viral RNA detection

RNA was extracted from sputum samples in TRIzol per manufacturer’s instructions. H7 hemagglutinin (HA) and N9 neuraminidase (NA) genes were detected by fluorescence reverse transcription (RT) PCR Detection kits (BioPerfectus Technologies, Taizhou, Jiangsu Province, China) provided by Nanjing CDC on the ABI 7500 (Applied Biosystems). Primers and protocols were prepared according to those provided by the WHO Collaborating Center in Beijing [7].

### Serum cytokine/chemokine assays

Frozen sera were thawed for cytokine/chemokine measurements using the Human Magnetic Cytokine/Chemokine Bead Panel –15 Plex (Millipore Corporation, Billerica, MA, USA) on the MAGPIX instrument (Luminex Corporation, Austin, TX, USA). The multiplex assay measures 15 serum cytokines, chemokines, and other immune biomarkers (GM-CSF, TNF-α, IFN-γ, IL-1RA, IL-1β, IL-2, IL-4, IL-6, IL-8, IL-10, IL-12P70, IL-17A, IP-10, MCP-1, and sCD40L), per manufacturer’s instructions.

### H7-specific binding antibodies

ELISA was conducted to measure H7 HA-specific IgG, IgA, and IgM responses in H7N9-infected patients. Briefly, 96-well flat-bottom plates were coated with recombinant H7 HA antigen of H7N9 A/Zhejiang/U01/2013, which was produced from DNA vaccine transfected 293T cells (-Haiyuan Protein Biotech, Inc., Taizhou, China) [28]. Plates were incubated with 100 µl horseradish peroxidase (HRP)-conjugated anti-human IgG, IgA, or IgM (Southern Biotech) and developed with 3,3,5,5-tetramethylbenzidine (TMB) solution (Sigma, St. Louis, MO). Data were presented as the OD value (for daily IgG) or titer of IgG, IgA, and IgM. The end titration titer was determined as the highest serum dilution that had an OD reading twice above that of the negative control serum.

### Influenza H7 pseudotyped virus production and neutralization assays

Levels of H7 HA-specific protective antibodies were measured using a pseudotyped virus system, as previously reported [29]–[31]. 293T cells with 80% confluence in 10 cm dishes were co-transfected with 1.2 µg HA-expressing plasmid (pJW4303/H7-HA ZJU01.wt), 0.3 µg NA-expressing plasmid (pJW4303/N9-NA SH02.wt), 13.5 µg HIV-1 replicon DNA (pNL4-3.LucR-E-), with deletion of envelope gene and insertion of the firefly luciferase gene in the Nef-encoding region, and 75 µl PEI (1 mg/ml) transfection reagent (Polysciences, Eppelheim, Germany) in 750 µl DMEM. Six hours later, the transfected cell culture was changed to freshly-made FBS-free DMEM medium. The supernatant was harvested 72 hours later, filtered and treated with Trypsin-TCPK (Sigma) at a final concentration of 40 µg/ml, then stopped with Trypsin inhibitor (Sigma) at 10 µg/ml. The median tissue culture infectious dose (TCID50) of H7 pseudovirus was titrated in 293A cells and stored at −80°C until use.

A neutralization assay was conducted using the above pseudotyped virus. Briefly, serial dilutions of patient serum samples (in duplicate) were incubated with H7 pseudovirus (200 TCID50 in 50 µl growth medium per well) in a 96-well cell culture plate; 293A cells (1×10^4^), in 100 µl growth medium, were added to each well. After incubation, cells were lysed and RLU was measured and 50% inhibitory dose (ID50) was determined.

### Data analysis

Student’s t-test was used to determine statistical significance of peak cytokine levels in patient sera. Spearman’s Rank-Order Correlation was used to determine the correlation between fever and cytokine levels in patients infected with H7N9.

## Results

**Figure 1:**
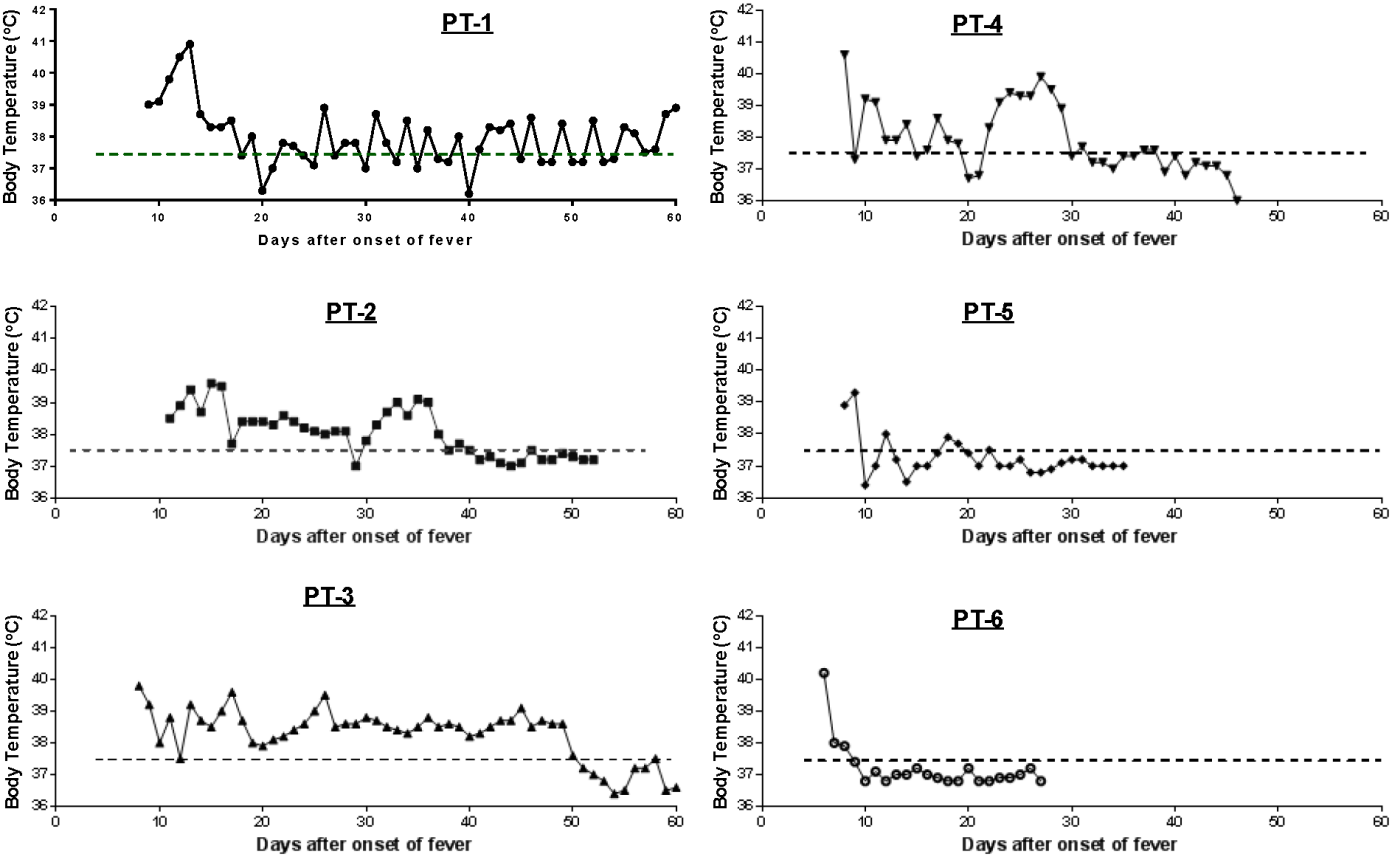
Daily temperature monitoring of H7N9 patients after admission. Daily body temperature of individual patients (PT-1 to PT-6) was recorded. The dashed line in each graph indicates the normal body temperature, 37.5°C, as the cutoff for fever.

During the initial H7N9 outbreak in the spring of 2013, six patients with confirmed H7N9 infection were admitted to the Nanjing Drum Tower Hospital (Table 1) [3], [32]. Four (66%) had a reported history of poultry contact and most had early onset upper respiratory symptoms. All had fever and were admitted to the hospital after varying lengths of fever (six to 11 days). Within the first week of admission, elevated body temperature, ranging from 38.5°C to 41°C, was observed in all six patients, and subsequently returned to normal levels following antiviral therapy and other treatment (Fig. 1). All six patients survived during the study period; four were discharged within the first 1–2 months of admission and two stayed longer than two months (Table 1). According to the medical records, Patient (Pt) #1 also had a co-morbidity of chronic hepatitis B virus infection and Pt #3 had resected thyroid carcinoma and leukocytosis, furthermore, both patients also had the complication with bacterial pneumonia, which contributed to their longer hospitalization after clearance of H7N9 infection.

**Figure 2:**
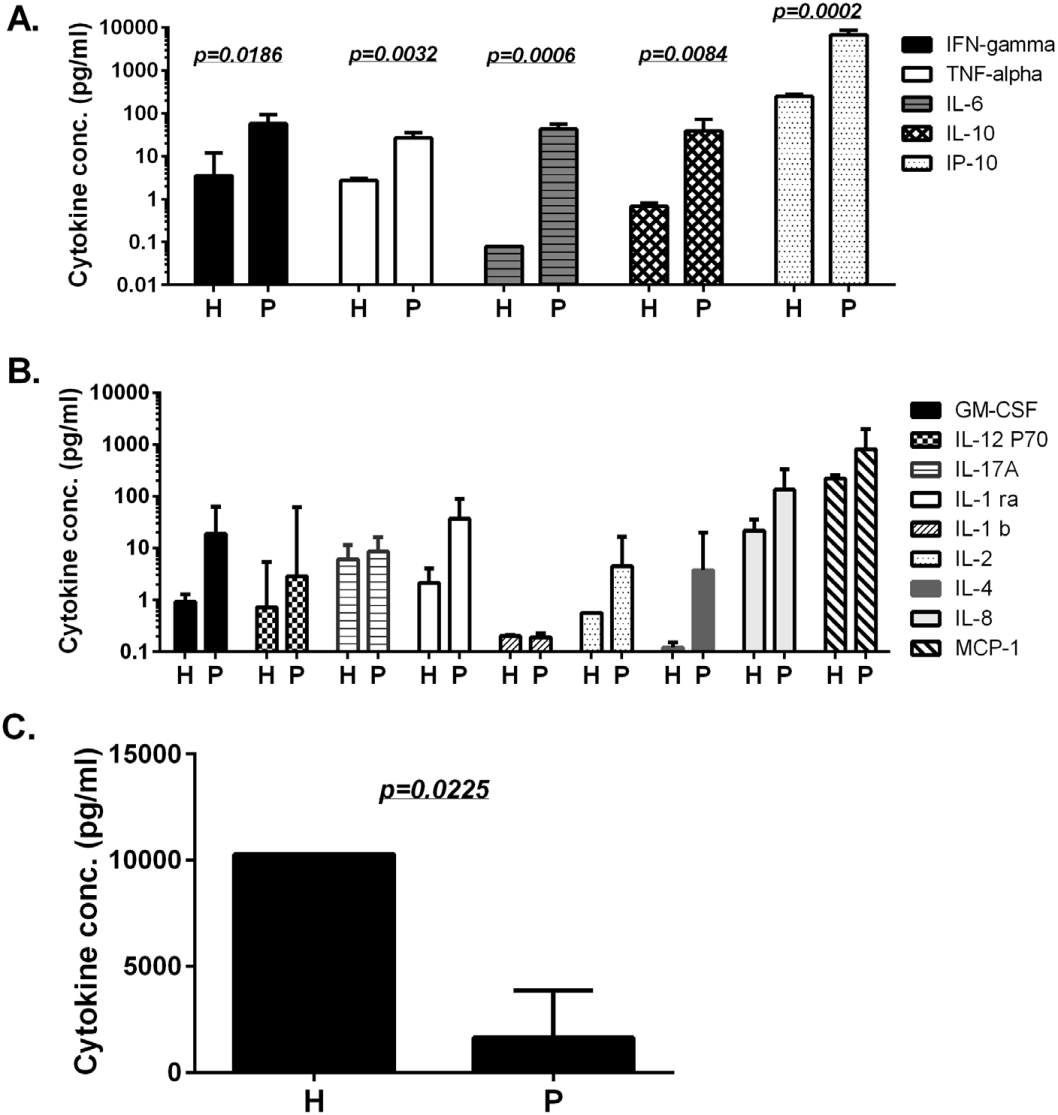
The peak serum cytokine and chemokine levels in H7N9 patients and healthy donors. “P” or “H” under each column indicates “H7N9 patients” or “healthy donors”, respectively. Three patterns of cytokine/chemokine profiles are shown: significantly elevated (A), elevated but not significant (B) or significantly suppressed (C) in H7N9-infected patients when compared to healthy donors. Each bar represents the geometric mean of each serum cytokine/chemokine concentration with standard error of in six patients or eight healthy donors. Statistical significance is indicated when the *p* value is less than −0.05between the patient and healthy donor groups.

Simultaneous measurement of serum cytokine profiles during the acute phase of H7N9 infection showed five were significantly elevated in H7N9-infected patients compared to healthy human controls (Fig. 2A). The other nine cytokines and chemokines were also elevated in H7N9-infected patients but not significantly (p>0.05) (Fig. 2B). In contrast, sCD40L serum levels were significantly suppressed in H7N9-infected patients (Fig. 2C).

**Figure 3:**
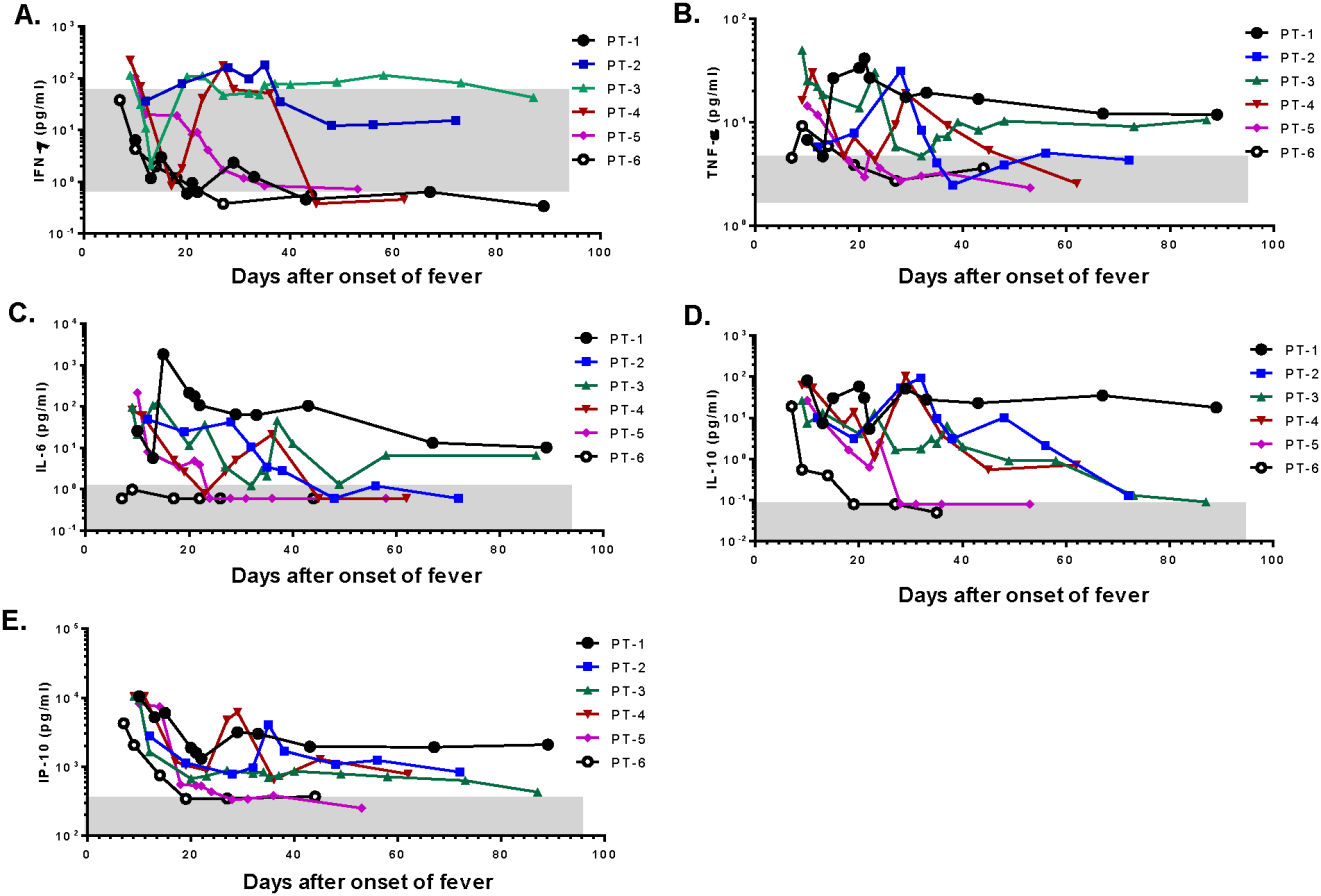
The kinetics of significantly elevated cytokines in H7N9-infected patients after admission. The kinetics of five significantly elevated serum cytokine concentrations is plotted for individual patients (PT-1–PT-6) during the time of hospitalization: IFN-γ (A), TNF-α (B), IL-6 (C), IL-10 (D), and IP-10 (E). The grey box in each graph indicates the relevant cytokine concentration range (geometric mean ± standard deviation) in normal healthy donors.

**Figure 4:**
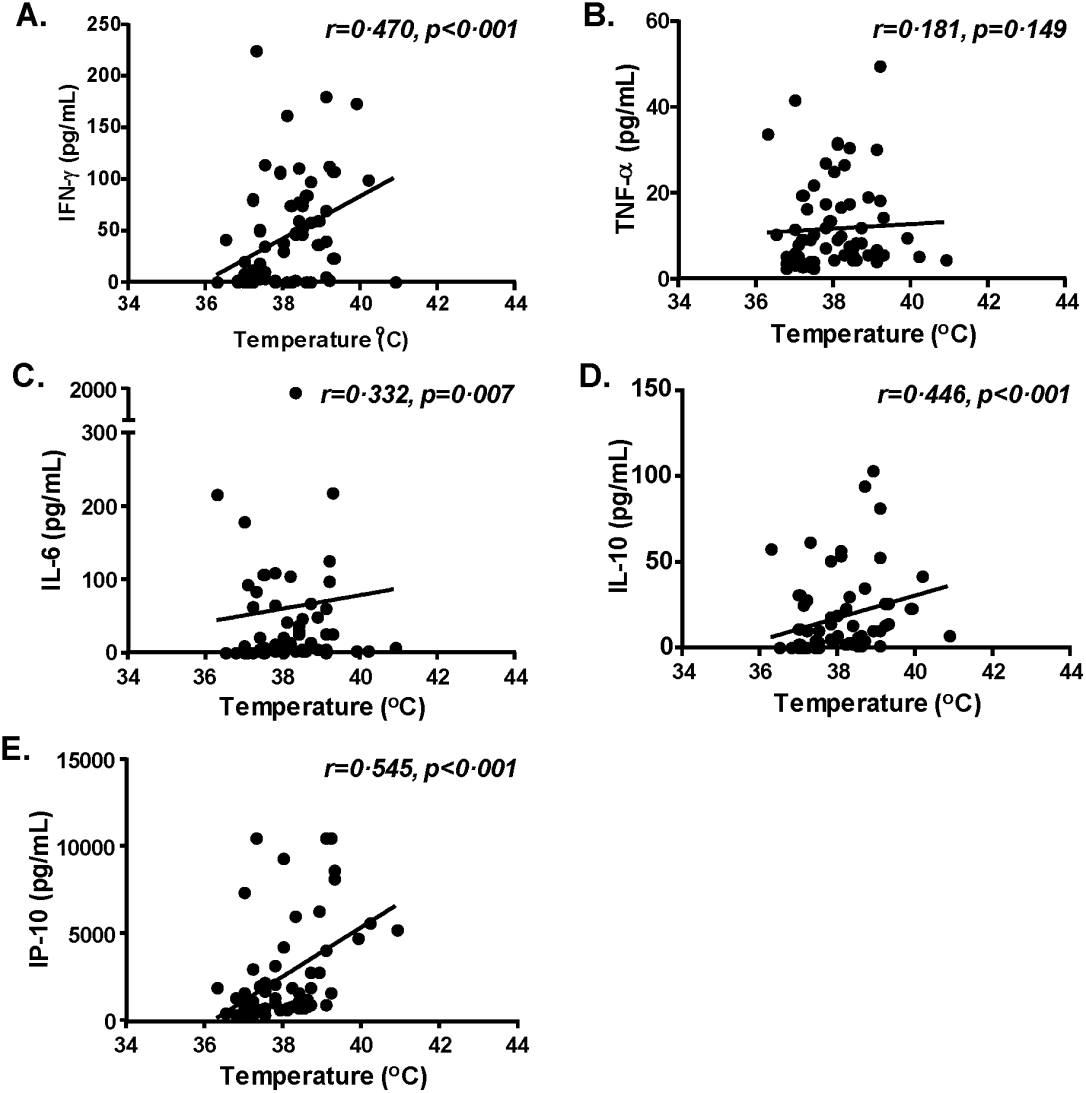
The relationship between significantly elevated serum cytokine levels and fever in H7N9-infected patients. Correlation between body temperature and each of the significantly elevated cytokines: IFN-γ (A), TNF-α (B), IL-6 (C), IL-10 (D), and IP-10 (E) were analyzed. The solid line in each graph represents the trend line of correlation. Each dot represents one patient at one time point.

Two patients (#1 and #3), who experienced prolonged clinical symptoms and a longer hospital stay, showed persistently elevated TNFα, IL-6, IL-10, and IP-10 (Fig. 3B–3E); furthermore, one patient (#3) also had elevated IFNγ for an extended period of time (Fig. 3A). On the other hand, two patients (#5 and #6), who had the quickest recovery and the shortest hospital stays, demonstrated a quick return to normal of all elevated cytokines. One patient (#6), who was discharged first among this cohort, did not have elevated serum IL-6 (Fig. 3C). Further analysis on temperature and cytokine levels from the same day showed that among the five significantly elevated cytokines, four (IFNγ, IL-6, IL-10, and IP-10) correlated with increases in body temperature (Fig. 4A, 4C–4E); no such relationship was observed for TNFα (Fig. 4B).

**Figure 5:**
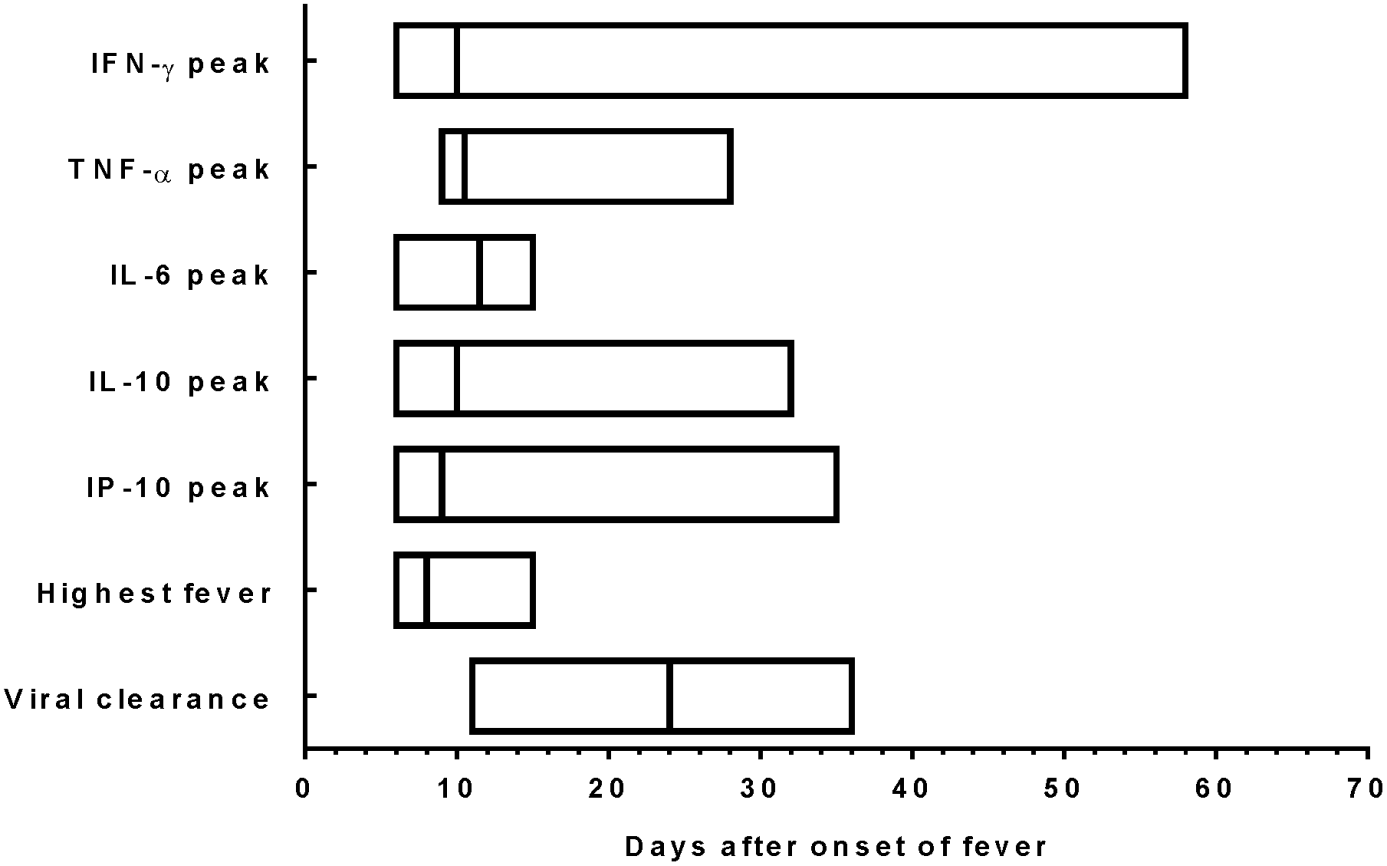
The time frame of significantly elevated serum cytokines at their peak levels, highest fever-, and clearance of H7N9 virus, as days (median and range) when IFN-γ, TNF-α, IL-6, IL-10, and IP-10 are at peak levels and days (median and range) when patients had the highest fever during hospitalization. Viral clearance is the time point when H7N9 viral RNA became undetectable.

The temporal relationship between viral clearance, highest fever, and peak major serum cytokines is shown in Fig. 5. It took a median of 24 days (range 11–36 days) to clear the H7N9 virus based on RT-PCR results of sputum (Fig. 5). The highest fever elevation occurred in the first 15 days of fever onset. Significantly elevated serum cytokines mostly occurred before complete clearance of H7N9. Peak IFNγ levels also occurred quite early although for some patients, peak occurred at a later time.

**Figure 6:**
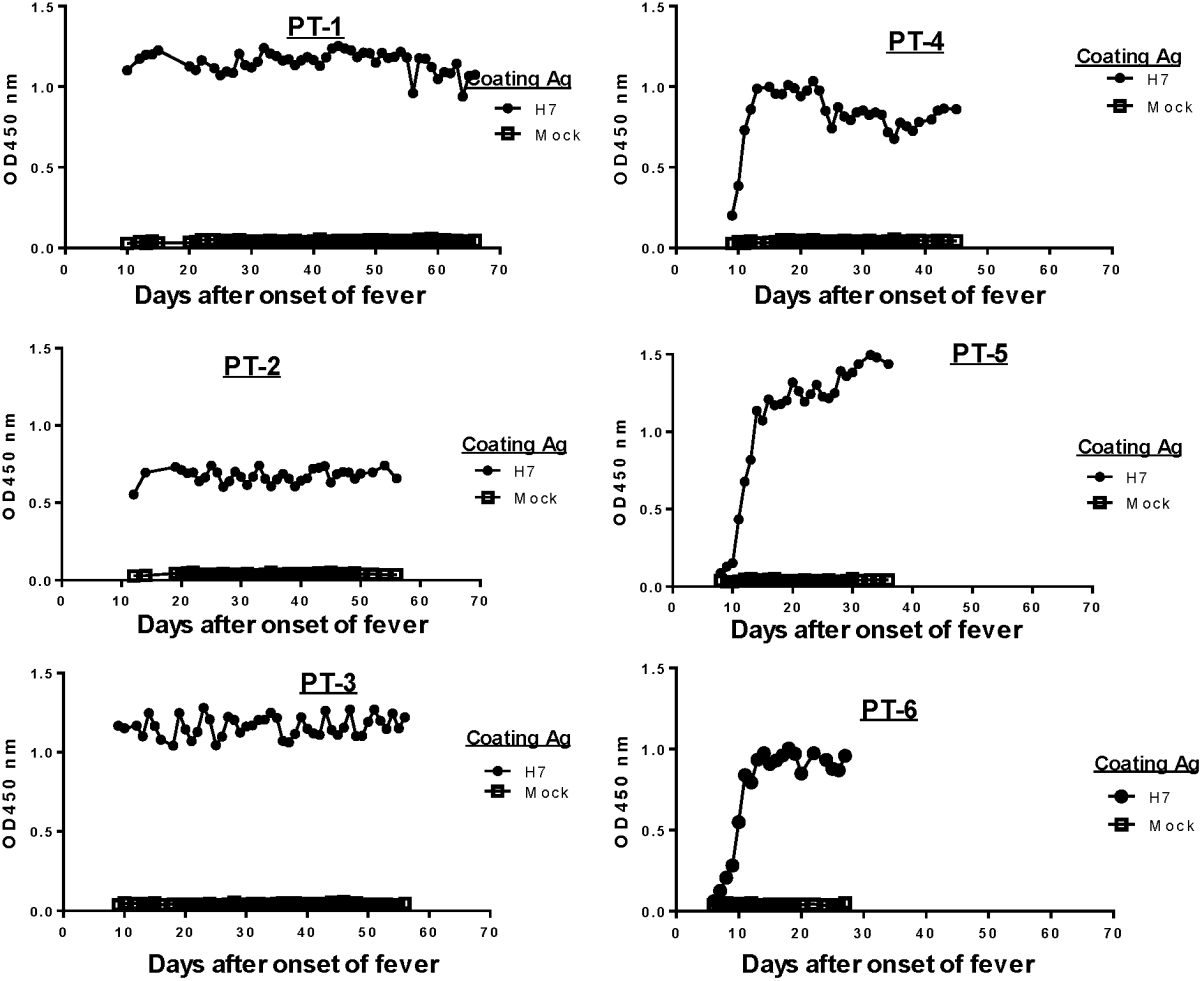
Daily H7 HA-specific IgG responses in H7N9-infected patients during hospitalization. ELISA OD values for daily antibody responses against H7 HA antigen in individual patients (PT-1–PT-6) with fixed sera dilution at 1∶500.

Serum antibody responses specific to the HA antigen of H7N9 were monitored daily from the day of admission until the time of discharge or at end of the current study period (day 60 after onset of fever) (Fig. 6). Three patients (#1–#3) had clearly elevated H7 HA-specific antibodies in their sera on the day of admission while three other patients (#4–#6) showed a daily increase in H7 HA-specific antibodies starting on the day of admission (Fig. 6). This finding suggested that the first three patients may have had a longer period of H7N9 infection before being admitted although, based on the onset of fever, the length of their pre-admission period was relatively similar to the other three patients (Table 1). It is possible that these patients may have had other clinical symptoms before developing fever so the onset of viral infection may have been longer before their admission. Interestingly, Patients #1–#3 also had a prolonged clinical course and stayed in the hospital longer (beyond Day 50 from onset of fever) compared with the other three patients (with stays between 28–46 days after onset of fever) (Fig. 1). Early hospitalization with specific treatment may contribute to a better clinical outcome.

**Figure 7:**
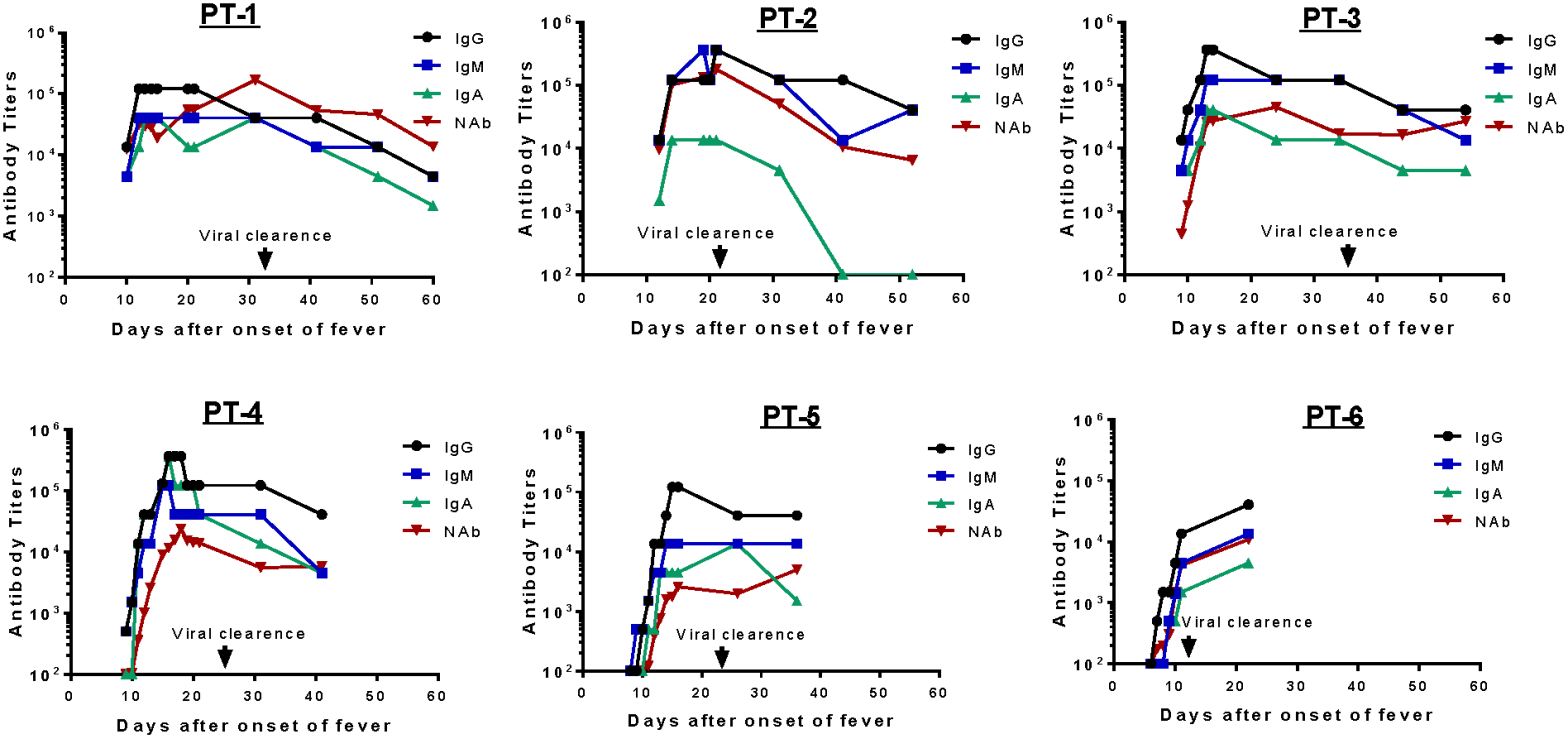
Kinetics of H7 HA-specific IgG, IgM, IgA and neutralizing antibody (NAb) responses in H7N9-infected patients during hospitalization. H7 HA-specific IgG, IgM, and IgA titers and NAb titers against H7 pseudovirus were measured at select time points in individual patients (PT-1–PT-6) after hospitalization. The time of viral clearance was marked when H7N9 viral RNA became undetectable.

Detailed analysis was conducted to determine isotype specificity of H7 HA antibody responses in infected patients (Fig. 7). For this analysis, antibody titers were determined by the end titration of serum against H7 HA antigen coated in ELISA plates; daily titers were determined only within the first 20 days of fever onset followed by titers determined every 10 days for the remaining period of hospitalization. The same two subgroups were observed as in the early days after admission. Patients #1–#3 had an H7 HA-specific IgG titer between 1∶10^4^ and 1∶10^5^, while the other three patients had low or below detectable levels of H7 HA-specific IgG responses. In the latter subgroup, H7 HA-specific IgG titers rose daily from the day of admission and, within one week, reached the same peak level IgG as the first three patients. Because the end titration titer approach was used in this analysis, H7 HA-specific IgG titers continued to rise even in the first three patients, providing evidence that these patients were also newly infected and might have a longer pre-admission infection course.

Further measurement of serum H7 HA-specific IgM (blue curve) and IgA (green curve) antibody responses revealed similar kinetics as observed for IgG (black curve) (Fig. 7). Several important findings are noted. First, IgM and IgA rose almost at the same time as IgG, which was not expected. Second, H7 HA-specific IgA titer responses were lower than IgG titers in most patients but IgM titers were similar to IgG responses. Third, after maintaining peak titers for no more than 20–30 days from the onset of fever, levels for all antibody isotypes (IgG, IgM, and IgA) started to drop. Typically, the magnitude of titer drop is about one to two logs. Because the current report only focused on acute immune responses, long-term H7 HA-specific antibody responses are currently unknown.

Neutralizing antibodies (red curve) against the H7N9 virus were measured in the current study (Fig. 7) using a pseudotyped virus system. Neutralizing antibodies developed at a later time point than IgG binding antibodies in four patients except #1 and #2 who had higher level binding antibodies early in the study. However, the delay in developing neutralizing antibodies was only a few days. Neutralizing antibody titers are, in general, lower than IgG binding antibodies as expected but it may also reflect the difference in assay methods. However, the trends of HA-specific IgG and neutralizing antibody kinetics were very similar. Although not all H7 HA IgG are protective, the levels of HA-specific IgG titers in patient sera may be useful to predict the levels of protective antibody. Interestingly, neutralizing antibody levels did not drop over time in four patients (#3–#6). This may suggest that functional antibodies are high affinity antibodies, which are products of mature B cells or plasma cells. Levels of these cells may be better maintained over time compared to less mature B cells.

## Discussion

H7N9 is a novel emerging avian influenza infection with a high human mortality rate. Innate immune responses play a key role in the pathogenesis and control of influenza infection. In the current study, we employed updated assay methodology to investigate serum levels of 15 key cytokines, chemokines, and immune modulators in H7N9-infected patients. Five (IL-6, IP-10, IL-10, IFNγ, and TNFα) were significantly elevated in H7N9-infected patients compared to age-matched healthy individuals. Although reports have demonstrated that the cytokine responses could be related to H7N9 pathogenicity in infected mice[33], [34], there are only a few reports about cytokine responses in infected patients. Our data are consistent but not identical to findings from these reports in the literature that studied cytokine and chemokine levels in H7N9-infected patients[8], [35]–[37]. In one study, IL-6 and IP-10 were significantly elevated in H7N9-infected patients, but IL-10, IFNγ and TNFα were not when compared to healthy controls [8]. On the other hand, that study showed a significant elevation of IL-2 and IL-17, which were also elevated in our study but not significantly [8]. Han et al. demonstrated that the fatal outcomes and disease severity were significantly associated with high TNFα levels in sera while the degree of liver damage were significantly correlated with their serum levels of Th2 cytokines IL-4 and IL-9 [36] whiles others demonstrated that the levels of serum IL-6, IL-8, C-reactive protein and MIP-1β were associated with the diseases severity [35], [37]. Because different commercial cytokine detection kits were used in various studies, some discrepancies are not unexpected, but data from these studies confirm that increases of IL-6 and IP-10 are prominent in H7N9-infected patients.

Measurement of serum cytokine profiles in other avian influenza infection, such as H5N1 or the 2009 pandemic H1N1 influenza (pH1N1), has been reported in a few studies, but highly sensitive and high-throughput technology was not used to directly measure a large panel of cytokines in acute influenza infection. However, a strong correlation between elevation of IL-6 and IL-10 and severe pH1N1 infection was noted [38]. In a separate study, IL-6, TNFα, IL-8, and IL-15 were significantly higher in the group with severe pH1N1 infection when a panel of nine serum cytokines was analyzed [39]. IL-10 was not included in this study. The role of IL-6, was further confirmed when IL-6 serum concentrations were significantly elevated in patients who required critical care support compared to patients who did not [40].

IL-6, IFNγ, and TNFα were also elevated in fatal human avian influenza H5N1 infection [41]. Serum IL-6, IL-10, and IFNγ continued to increase during the clinical course; IP-10 and MCP-1 remained at a high level from the beginning of infection [8]. In an *in vitro* study using human primary alveolar and bronchial epithelial cells, H5N1 viruses were more potent inducers of IL-6, IP-10, RANTES, and IFNβ compared to seasonal human H1N1 viruses [42].

Taken all together, it appears that IL-6 is elevated in all types of influenza infection and IP-10 is another potentially important cytokine in influenza infection. IL-6 is a proinflammatory cytokine of innate immunity [43], which is secreted by T cells and macrophages to stimulate immune responses during infection. IL-6 also supports the growth of B cells and is antagonistic to regulatory T cells. Consistent with clinical reports, IL-6 was also increased during the host response to pH1N1 infection in mice, supporting the idea that IL-6 is an indicator of disease severity for influenza infection.

IP-10 (Interferon gamma-induced protein 10) is a chemokine produced in macrophage and dendritic cells. IP-10 may serve several roles, such as chemoattraction for monocytes/macrophages, T cells, NK cells, and promotion of T cell adhesion to endothelial cells. High levels of IP-10 were found in hepatitis C virus (HCV)- or human immunodeficiency virus type 1 (HIV-1)-infected patients who did not respond well to anti-viral treatment [44]–[46].

Three other significantly elevated cytokines in our study have a wide range of biological functions. IFNγ is a cytokine of innate and adaptive immunity that activates macrophages, differentiates Th1 from T cells, inhibits the Th17 pathway, and controls intracellular pathogens [47]. TNFα is a cytokine of innate immunity and its principal cellular targets and biological effects include activation of endothelial cells, neutrophil activation, and fever, among others. IL-10 is an anti-inflammatory cytokine with pleiotropic effects in immunoregulation and inflammation; it downregulates the expression of Th1 cytokines, class II MHC antigens, and co-stimulatory molecules on macrophages. IL-10 also enhances B cell survival, proliferation, and antibody production. Because IFNγ, TNFα, and IL-10 have a broad spectrum of biological functions, their role in acute H7N9 infection and their impact on the final clinical outcome remains to be elucidated.

Influenza antigen-specific antibody responses are critical in controlling influenza transmission and infection. A lack of pre-existing antibody responses in the human population is a key factor for the fast spread of a novel pandemic influenza at a global scale. Influenza infection is self-limiting to most healthy individuals due to a quick development of antibody responses. However, there is a lack of information on how specific antibody responses are developed in patients who are infected by a novel influenza that has not previously circulated in the human population. Annual seasonal influenza immunization is a “boost” to pre-existing immunity to seasonal influenza in human populations generated either by subclinical exposure to seasonal influenza in previous years or via early flu immunizations. In the development of vaccines against novel H5N1 viruses, two times immunization is needed to achieve protective antibody responses [48]. A higher vaccine dose or strong adjuvant may enhance the immunogenicity of H5N1 vaccines but may not be as effective as twice immunization. It is important to learn how antibody responses are generated against an acute influenza infection in a naïve host who has not been exposed to a subtype of influenza virus such as H7N9.

In the current study, H7 HA-specific antibody responses were detected soon after onset of fever and continued to rise until reaching peak levels within two weeks. Protective antibody responses appeared at only 2–3 days after the appearance of binding antibody responses. The most striking result is the appearance of H7 HA-specific IgM and IgA antibody responses at the same time as IgG responses; furthermore, slopes of antibody response curves were similar among three isotypes. The only difference was that peak level IgA responses were lower than IgM and IgG peak titers. H7 HA-specific antibody titers of all three major isotypes started to decrease shortly after reaching peak levels but were still positive at the end of this study.

There is limited information available on patterns of antibody responses during acute avian influenza infection. While traditional immunology theory would suggest that IgM antibody responses occur before IgG responses in a new viral infection, two recent reports described early detection of IgA responses in acute HIV-1 infection [49], [50]. It will be useful to learn how an acute live virus infection is effective in stimulating simultaneous IgM and IgG responses, which may help design better vaccines to quickly elicit powerful and protective IgG antibody responses, without the need to first elicit IgM responses.

## References

[1] Lam TT, Wang J, Shen Y, Zhou B, Duan L, et al (2013) The genesis and source of the H7N9 influenza viruses causing human infections in China. Nature 502: 241–244. 23965623 10.1038/nature12515 PMC3801098

[2] Koopmans M, de Jong MD (2013) Avian influenza A H7N9 in Zhejiang, China. Lancet 381: 1882–1883. 23628442 10.1016/S0140-6736(13)60936-8

[3] Wu C, Huang R, Chen J, Gu Q, Zhu B, et al (2013) Avian Influenza A(H7N9) Virus Screening in Patients with Fever and Flu-Like Symptoms in a Tertiary Hospital in an Area with Confirmed Cases. PLoS ONE 8: e82613. 24367529 10.1371/journal.pone.0082613 PMC3867373

[4] Wu S, Wu F, He J (2013) Emerging risk of H7N9 influenza in China. Lancet 381: 1539–1540. 10.1016/S0140-6736(13)60767-9 PMC7137082 23602315

[5] Bai T, Zhou J, Shu Y (2013) Serologic study for influenza A (H7N9) among high-risk groups in China. N Engl J Med 368: 2339–2340. 23718151 10.1056/NEJMc1305865

[6] Gao HN, Lu HZ, Cao B, Du B, Shang H, et al (2013) Clinical findings in 111 cases of influenza A (H7N9) virus infection. N Engl J Med 368: 2277–2285. 23697469 10.1056/NEJMoa1305584

[7] Gao R, Cao B, Hu Y, Feng Z, Wang D, et al (2013) Human infection with a novel avian-origin influenza A (H7N9) virus. N Engl J Med 368: 1888–1897. 23577628 10.1056/NEJMoa1304459

[8] Chi Y, Zhu Y, Wen T, Cui L, Ge Y, et al (2013) Cytokine and Chemokine Levels in Patients Infected With the Novel Avian Influenza A (H7N9) Virus in China. J Infect Dis 208: 1962–1967. 23990573 10.1093/infdis/jit440

[9] Belser JA, Gustin KM, Pearce MB, Maines TR, Zeng H, et al (2013) Pathogenesis and transmission of avian influenza A (H7N9) virus in ferrets and mice. Nature 501: 556–559. 23842497 10.1038/nature12391 PMC7094885

[10] Fouchier RA, Kawaoka Y, Cardona C, Compans RW, Fouchier RA, et al (2013) Avian flu: Gain-of-function experiments on H7N9. Nature 500: 150–151. 23925229 10.1038/500150a

[11] Han J, Niu F, Jin M, Wang L, Liu J, et al (2013) Clinical presentation and sequence analyses of HA and NA antigens of the novel H7N9 viruses. Emerging Microbes & Infection 2: e23. 10.1038/emi.2013.28 PMC3675404 26038463

[12] Liu X, Li T, Zheng Y, Wong K-W, Lu S, et al (2013) Poor responses to oseltamivir treatment in a patient with influenza A (H7N9) virus infection. Emerging Microbes & Infections 2: e27. 26038464 10.1038/emi.2013.30 PMC3675405

[13] Richard M, Schrauwen EJ, de Graaf M, Bestebroer TM, Spronken MI, et al (2013) Limited airborne transmission of H7N9 influenza A virus between ferrets. Nature 501: 560–563. 23925116 10.1038/nature12476 PMC3819191

[14] Watanabe T, Kiso M, Fukuyama S, Nakajima N, Imai M, et al (2013) Characterization of H7N9 influenza A viruses isolated from humans. Nature 501: 551–555. 23842494 10.1038/nature12392 PMC3891892

[15] Wen Y-M, Klenk H-D (2013) H7N9 avian influenza virus-search and re-search. Emerging Microbes & Infections 2: e18. 26038459 10.1038/emi.2013.18 PMC3636592

[16] Xiong X, Martin SR, Haire LF, Wharton SA, Daniels RS, et al (2013) Receptor binding by an H7N9 influenza virus from humans. Nature 499: 496–499. 23787694 10.1038/nature12372

[17] Yang F, Wang J, Jiang L, Jin J, Shao L, et al (2013) A fatal case caused by novel H7N9 avian influenza A virus in China. Emerging Microbes & Infections 2: e19. 26038460 10.1038/emi.2013.22 PMC3636593

[18] Yu X, Zhang X, He Y, Wu H, Gao X, et al (2013) Mild infection of a novel H7N9 avian influenza virus in children in Shanghai. Emerging Microbes & Infections 2: e41. 26038475 10.1038/emi.2013.41 PMC3820982

[19] Zhou J, Wang D, Gao R, Zhao B, Song J, et al (2013) Biological features of novel avian influenza A (H7N9) virus. Nature 499: 500–503. 23823727 10.1038/nature12379

[20] Zhu Z, Yang Y, Feng Y, Shi B, Chen L, et al (2013) Infection of inbred BALB/c and C57BL/6 and outbred Institute of Cancer Research mice with the emerging H7N9 avian influenza virus. Emerging Microbes & Infections 2: e50. 26038485 10.1038/emi.2013.50 PMC3821289

[21] WHO (2013) Human infection with avian influenza A(H7N9) virus–update. WHO.

[22] Hvistendahl M, Normile D, Cohen J (2013) Influenza. Despite large research effort, H7N9 continues to baffle. Science 340: 414–415. 23620023 10.1126/science.340.6131.414

[23] Li Q, Zhou L, Zhou M, Chen Z, Li F, et al. (2013) Preliminary Report: Epidemiology of the Avian Influenza A (H7N9) Outbreak in China. N Engl J Med.

[24] Yu H, Cowling BJ, Feng L, Lau EH, Liao Q, et al (2013) Human infection with avian influenza A H7N9 virus: an assessment of clinical severity. Lancet 382: 138–145. 23803487 10.1016/S0140-6736(13)61207-6 PMC3801178

[25] Qiu C, Huang Y, Zhang A, Tian D, Wan Y, et al (2013) Safe pseudovirus-based assay for neutralization antibodies against influenza A(H7N9) virus. Emerg Infect Dis 19: 1685–1687. 24047684 10.3201/eid1910.130728 PMC3810762

[26] Yu L, Wang Z, Chen Y, Ding W, Jia H, et al (2013) Clinical, Virological, and Histopathological Manifestations of Fatal Human Infections by Avian Influenza A(H7N9) Virus. Clinical Infectious Diseases 57: 1449–1457. 23943822 10.1093/cid/cit541

[27] Yang S, Chen Y, Cui D, Yao H, Lou J, et al. (2013) Avian-Origin Influenza A(H7N9) Infection in Influenza A(H7N9)-Affected Areas of China: A Serological Study. J Infect Dis. 10.1093/infdis/jit430 23935201

[28] Zhang L, Jia N, Li J, Han Y, Cao W, et al. (2014) Optimal designs of an HA-based DNA vaccine against H7 subtype influenza viruses. Hum Vaccin Immunother 10. 10.4161/hv.28795 PMC4186056 25424804

[29] Yang ZY, Wei CJ, Kong WP, Wu L, Xu L, et al (2007) Immunization by avian H5 influenza hemagglutinin mutants with altered receptor binding specificity. Science 317: 825–828. 17690300 10.1126/science.1135165 PMC2367145

[30] Wang S, Hackett A, Jia N, Zhang C, Zhang L, et al (2011) Polyvalent DNA vaccines expressing HA antigens of H5N1 influenza viruses with an optimized leader sequence elicit cross-protective antibody responses. PLoS One 6: e28757. 22205966 10.1371/journal.pone.0028757 PMC3244406

[31] Almansour I, Chen H, Wang S, Lu S (2013) Cross reactivity of serum antibody responses elicited by DNA vaccines expressing HA antigens from H1N1 subtype influenza vaccines in the past 30 years. Hum Vaccin Immunother 9. 10.4161/hv.25735 PMC3906389 23884239

[32] Wang Y, Zhang Y, Wang H, Zhou Z, Zhou Z, et al (2013) Sharing insights and H7N9 patient clinical data. Am J Respir Crit Care Med 188: 115–117. 23815729 10.1164/rccm.201305-0936LE

[33] Zhao G, Liu C, Kou Z, Gao T, Pan T, et al (2014) Differences in the pathogenicity and inflammatory responses induced by avian influenza A/H7N9 virus infection in BALB/c and C57BL/6 mouse models. PLoS One 9: e92987. 24676272 10.1371/journal.pone.0092987 PMC3968029

[34] Mok CK, Lee HH, Chan MC, Sia SF, Lestra M, et al. (2013) Pathogenicity of the novel A/H7N9 influenza virus in mice. MBio 4. 10.1128/mBio.00362-13 PMC3705449 23820393

[35] Shen Z, Chen Z, Li X, Xu L, Guan W, et al. (2013) Host immunological response and factors associated with clinical outcome in patients with the novel influenza A H7N9 infection. Clin Microbiol Infect. 10.1111/1469-0691.12505 24350809

[36] Han J, Zhang N, Zhang P, Yang C, Jin M, et al. (2014) Th2-type inflammation under conditions of pre-existing chronic disease is associated with liver damage in patients with avian influenza H7N9 virus. Microbes Infect. 10.1016/j.micinf.2014.04.002 24769417

[37] Wang Z, Zhang A, Wan Y, Liu X, Qiu C, et al (2014) Early hypercytokinemia is associated with interferon-induced transmembrane protein-3 dysfunction and predictive of fatal H7N9 infection. Proc Natl Acad Sci U S A 111: 769–774. 24367104 10.1073/pnas.1321748111 PMC3896201

[38] Yu X, Zhang X, Zhao B, Wang J, Zhu Z, et al (2011) Intensive cytokine induction in pandemic H1N1 influenza virus infection accompanied by robust production of IL-10 and IL-6. PLoS One 6: e28680. 22174866 10.1371/journal.pone.0028680 PMC3235144

[39] Hagau N, Slavcovici A, Gonganau DN, Oltean S, Dirzu DS, et al (2010) Clinical aspects and cytokine response in severe H1N1 influenza A virus infection. Crit Care 14: R203. 21062445 10.1186/cc9324 PMC3220006

[40] Paquette SG, Banner D, Zhao Z, Fang Y, Huang SS, et al (2012) Interleukin-6 is a potential biomarker for severe pandemic H1N1 influenza A infection. PLoS One 7: e38214. 22679491 10.1371/journal.pone.0038214 PMC3367995

[41] To KF, Chan PK, Chan KF, Lee WK, Lam WY, et al (2001) Pathology of fatal human infection associated with avian influenza A H5N1 virus. J Med Virol 63: 242–246. 11170064 10.1002/1096-9071(200103)63:3<242::aid-jmv1007>3.0.co;2-n

[42] Chan MC, Cheung CY, Chui WH, Tsao SW, Nicholls JM, et al (2005) Proinflammatory cytokine responses induced by influenza A (H5N1) viruses in primary human alveolar and bronchial epithelial cells. Respir Res 6: 135. 16283933 10.1186/1465-9921-6-135 PMC1318487

[43] Korn T, Oukka M, Kuchroo V, Bettelli E (2007) Th17 cells: effector T cells with inflammatory properties. Semin Immunol 19: 362–371. 18035554 10.1016/j.smim.2007.10.007 PMC2839934

[44] Romero AI, Lagging M, Westin J, Dhillon AP, Dustin LB, et al (2006) Interferon (IFN)-gamma-inducible protein-10: association with histological results, viral kinetics, and outcome during treatment with pegylated IFN-alpha 2a and ribavirin for chronic hepatitis C virus infection. J Infect Dis 194: 895–903. 16960776 10.1086/507307

[45] Lagging M, Romero AI, Westin J, Norkrans G, Dhillon AP, et al (2006) IP-10 predicts viral response and therapeutic outcome in difficult-to-treat patients with HCV genotype 1 infection. Hepatology 44: 1617–1625. 17133471 10.1002/hep.21407

[46] Falconer K, Askarieh G, Weis N, Hellstrand K, Alaeus A, et al (2010) IP-10 predicts the first phase decline of HCV RNA and overall viral response to therapy in patients co-infected with chronic hepatitis C virus infection and HIV. Scand J Infect Dis 42: 896–901. 20608766 10.3109/00365548.2010.498019

[47] Miossec P, Korn T, Kuchroo VK (2009) Interleukin-17 and type 17 helper T cells. N Engl J Med 361: 888–898. 19710487 10.1056/NEJMra0707449

[48] Middleton D, Rockman S, Pearse M, Barr I, Lowther S, et al (2009) Evaluation of vaccines for H5N1 influenza virus in ferrets reveals the potential for protective single-shot immunization. J Virol 83: 7770–7778. 19457991 10.1128/JVI.00241-09 PMC2708649

[49] Tomaras GD, Ferrari G, Shen X, Alam SM, Liao H-X, et al. (2013) Vaccine-induced plasma IgA specific for the C1 region of the HIV-1 envelope blocks binding and effector function of IgG. Proceedings of the National Academy of Sciences. 10.1073/pnas.1301456110 PMC3670311 23661056

[50] Yates NL, Lucas JT, Nolen TL, Vandergrift NA, Soderberg KA, et al (2011) Multiple HIV-1-specific IgG3 responses decline during acute HIV-1: implications for detection of incident HIV infection. AIDS 25: 2089–2097. 21832938 10.1097/QAD.0b013e32834b348e PMC3667583

